# Functional training improves peak oxygen consumption and quality of life of individuals with heart failure: a randomized clinical trial

**DOI:** 10.1186/s12872-023-03404-7

**Published:** 2023-07-29

**Authors:** Daniela Meirelles do Nascimento, Karina Costa Machado, Patrícia Martins Bock, Marco Aurélio Lumertz Saffi, Livia Adams Goldraich, Anderson Donelli Silveira, Nadine Clausell, Beatriz D. Schaan

**Affiliations:** 1 Exercise Pathophysiology Laboratory, Hospital de Clínicas de Porto Alegre, Porto Alegre, RS Brazil; 2 Clinical Research Center, National Institute of Science and Technology for Health Technology Assessment (IATS) – CNPq/Brazil, Hospital de Clínicas de Porto Alegre, Rua Ramiro Barcelos, Porto Alegre, RS 2350 Brazil; 3 Faculdades Integradas de Taquara, Taquara, RS Brazil; 4 Hospital de Clínicas de Porto Alegre, Porto Alegre, RS Brazil; 5 Medical School, Universidade Federal do Rio Grande do Sul, Porto Alegre, RS Brazil

**Keywords:** Cardiac failure, Exercise, Cardiopulmonary exercise capacity, Health-related quality of life

## Abstract

**Background:**

Functional training may be an effective non-pharmacological therapy for heart failure (HF). This study aimed to compare the effects of functional training with strength training on peak VO_2_ and quality of life in individuals with HF.

**Methods:**

A randomized, parallel-design and examiner-blinded controlled clinical trial with concealed allocation, intention-to-treat and per-protocol analyses. Twenty-seven participants with chronic HF were randomly allocated to functional or strength training group, to perform a 12-week physical training, three times per week, totalizing 36 sessions. Primary outcomes were the difference on peak VO_2_ and quality of life assessed by cardiopulmonary exercise testing and Minnesota Living with Heart Failure Questionnaire, respectively. Secondary outcomes included functionality assessed by the Duke Activity Status Index and gait speed test, peripheral and inspiratory muscular strength, assessed by hand grip and manovacuometry testing, respectively, endothelial function by brachial artery flow-mediated dilation, and lean body mass by arm muscle circumference.

**Results:**

Participants were aged 60 ± 7 years, with left ventricular ejection fraction 29 ± 8.5%. The functional and strength training groups showed the following results, respectively: peak VO_2_ increased by 1.4 ± 3.2 (16.9 ± 2.9 to 18.6 ± 4.8 mL.kg^−1^.min^−1^; p time = 0.011) and 1.5 ± 2.5 mL.kg^−1^.min^−1^ (16.8 ± 4.0 to 18.6 ± 5.5 mL.kg^−1^.min^−1^; p time = 0.011), and quality of life score decreased by 14 ± 15 (25.8 ± 14.8 to 10.3 ± 7.8 points; p time = 0.001) and 12 ± 28 points (33.8 ± 23.8 to 19.0 ± 15.1 points; p time = 0.001), but no difference was observed between groups (peak VO_2_: p interaction = 0.921 and quality of life: p interaction = 0.921). The functional and strength training increased the activity status index by 6.5 ± 12 and 5.2 ± 13 points (p time = 0.001), respectively, and gait speed by 0.2 ± 0.3 m/s (p  time = 0.002) in both groups.

**Conclusions:**

Functional and strength training are equally effective in improving peak VO_2_, quality of life, and functionality in individuals with HF. These findings suggest that functional training may be a promising and innovative exercise-based strategy to treat HF.

**Trial registration:**

NCT03321682. Registered date: 26/10/2017.

**Supplementary Information:**

The online version contains supplementary material available at 10.1186/s12872-023-03404-7.

## Background

Reduced tolerance to exercise is a symptom of heart failure (HF) and it is associated with increased disability and mortality [1]. The sedentary lifestyle adopted by individuals with HF leads to reductions on peak oxygen consumption (VO_2_) and poor quality of life [2]. A recent meta-analysis of randomized clinical trials shows that exercise-based cardiac rehabilitation improves quality of life and functional capacity [3], supporting the recommendation of international guidelines that exercise training is useful to improve functional status, exercise performance, and quality of life in HF [4, 5].

Individuals with HF show a 30% decrease in their ability to perform daily activities compared to healthy individuals, which is attributed to reduced muscular mass and decreased VO_2_ [6]. Activities of daily living require a combination of endurance and strength, and aerobic training alone could not improve muscular strength [7]. Particularly, strength training does not usually represent the movements of routine daily activities, since it does not include exercises that require coordinated and multiplanar movement patterns or incorporate multiple joints and dynamic tasks [8]. In addition, impaired balance, mobility, and gait performance has been found in individuals with HF when compared with healthy controls [9], and have been strongly associated with quality of life [10].

In this context, functional training may be a potentially effective non-pharmacological therapy to increase physical function for individuals with HF. This modality of physical training consists of differentiated integrated movements of the body that involve joint acceleration and deceleration, stabilization, strength, and neuromuscular efficiency [11]. A recent systematic review of nine non-randomized clinical trials indicated that functional training could increase speed, muscle strength, power, flexibility, agility, balance, aerobic, and muscular endurance among athletes [12]. Functional training, also known as neuromotor exercise training, is recommended by the American College of Sports Medicine for apparently healthy adults of all ages [13]. However, the effectiveness of functional training in chronic diseases has not been established.

In general, studies on functional training focus on assessing functionality in older adults based on walking capacity [14], mobility [15], and prevention of falls [16]. Recent evidence suggests that combined aerobic and resistance training improve peak VO_2_, muscular strength and quality of life and must be considered as a component of care for individuals with HF with reduced left ventricular ejection fraction [17]. Although the association between cardiopulmonary capacity, strength muscle, and quality of life has been researched, the results have been divergent [9, 10]. Besides that, studies [14–16] related to functional training did not include peak VO_2_ as outcome, and some aspects—such as volume, performance patterns, and progression of this type of training – remain unknown. Thereby, we hypothesized that functional training improves peak VO_2_ and muscle strength, and it has a positive impact on the functionality and the quality of life of patients with HF. Therefore, the present study aimed to compare the effects of functional training with strength training on peak VO_2_, quality of life, functionality, muscular strength, endothelial function and lean body mass, and the safety of a functional training program for individuals with HF.

## Methods

This is a randomized, parallel-design, 1:1 ratio allocation, examiner-blinded controlled clinical trial with concealed allocation [18]. This study is in accordance with the Declaration of Helsinki and was approved by the Scientific Ethics and Research Committee of the institution (no. 20170291) and the Brazilian government’s registry of scientific studies (Plataforma Brasil, no. 69314017.8.0000.5327). The trial was previously registered on Clinicaltrials.gov (NCT03321682). All participants provided written informed consent before participation, according to resolution 466/2012. This clinical trial is reported in accordance with CONSORT guidelines [19].

### Participants

Individuals of both genders, aged ≥ 40 years, were recruited from the outpatient cardiology clinic of a tertiary public hospital in southern Brazil. The inclusion criteria were: clinically stable HF (ischemic and non-ischemic) for at least three months before randomization, diagnosed according to clinical records; New York Heart Association (NYHA) functional classes II-III, with slight or clear limitation of physical activity, respectively; left ventricular ejection fraction ≤ 45%; and optimized pharmacological treatment. Individuals were excluded if: they were enrolled in another clinical trial involving physical training protocols or in regular practice of physical exercise in the previous three months; they had decompensated HF; they presented acute myocardial infarction and/or cardiac surgery in the previous six months; they suffered from severe valvular heart diseases and/or uncontrolled cardiac arrhythmias; they had asymmetric septal hypertrophic cardiomyopathy with dynamic obstruction in the outflow pathway; they suffered from musculoskeletal disorders that would limit the execution of the exercise program; impaired cognitive status that would compromise the understanding and the execution of the study protocols.

### Training program

The participants were randomly allocated either to an experimental group (functional training group – FTG) or to an active comparator group (strength training group – STG). Each training consisted of 12 weeks, performed three times per week, totaling 36 sessions. The exercises were modified every 12 sessions and the participants were encouraged by the main researcher—a physical therapist—to exercise at high performance (time or number of repetitions) and to progressively increase the difficulty of each exercise. The exercise sessions were completed at the hospital clinical research center. Exercises and periodization model for both physical trainings were previously described [20].

### Outcomes

The outcomes were assessed by a blinded examiner at the end of the 12 weeks of both physical trainings. Baseline demographic and clinical information – age, gender, HF etiology, left ventricle ejection fraction, and NYHA functional class – were obtained from electronic health records.

### Measurement of primary outcomes

Peak VO_2_ was measured during cardiopulmonary exercise testing with expired gas analysis, performed on a treadmill (T2100, speed 0–22 km/h [0–13.5 mph], grade 0–26%, General Electric, Wisconsin, USA). A ramp protocol was used with a starting speed of 2.0 km/h or 2.5 km/h and a starting grade of 0%. Increments of 0.5 km/h per minute in speed and 1% per minute in grade were used to achieve fatigue within 8–12 min. During the test, gas exchanges were continuously measured breath-by-breath by a previously validated system (Quark CPET; COSMED, Rome, Italy). Peak VO_2_ (mL.kg^−1^.min^−1^) was set to the highest 20 s average value reached during the test. Maximality criteria, defined by a respiratory exchange ratio ≥ 1.05 [21], was achieved by all subjects in the study.

Quality of life was assessed by the Minnesota Living with Heart Failure Questionnaire, a disease-specific instrument. Total score ranges from zero to 105 points, with low scores reflecting a better health-related quality of life [22]. The minimal clinically important difference considered on the Minnesota Living with Heart Failure Questionnaire is five points [23].

### Measurement of secondary outcomes

Functionality was evaluated by the Duke Activity Status Index (DASI) and the gait speed test. The DASI ranges from zero to 58.2 points, with a higher score representing a better functional capacity [24]. A difference of two or more units on DASI score is considered clinically meaningful [25]. The gait speed test was performed in a 20 m-long corridor. The participants walked at their own pace, without running, and the time spent in the central 10 m of the corridor was determined. Then, the ratio between distance and time (meters/second) was calculated [26].

The hand grip dynamometer (JAMAR®, Sammons Preston, Inc., Bolingbook, IL, USA) was used to evaluate peripheral muscular strength [27]. Strength values were calculated in kilograms, and the average of three attempts for the dominant hand, performed with a one-minute interval between measures, was considered. Inspiratory muscular strength or maximal inspiratory pressure (cmH_2_O) were evaluated by manovacuometry [28]. At least three reproducible maneuvers were performed using a digital pressure manometer (MVD300 Microhard System*®*, Globalmed, Porto Alegre, Brazil). The highest value was recorded for data analysis, as long as it did not exceed the second highest value by 10%.

Non-invasive measurements of endothelial function were obtained by flow-mediated dilation (FMD) and nitroglycerine-induced vasodilation of the brachial artery using two-dimensional ultrasound equipment, in accordance with published guidelines [29] and always by the same trained operator. The FMD method involves ultrasound arterial imaging in two conditions: at rest (baseline) and during reactive hyperemia after a five-minute arterial occlusion. Nitroglycerine-induced vasodilation, an index of endothelium-independent vasodilation, was assessed five minutes after the administration of a single sublingual 0.4 mg dose of nitroglycerine. Both FMD and nitroglycerine-induced vasodilation were calculated as the percent change in peak vessel diameter from the baseline value by using [(peak diameter − baseline diameter) / baseline diameter] × 100 [30].

Lean body mass was evaluated based on the arm muscle circumference, which was obtained from arm circumference and tricipital skinfold measurements by using the tape measure and adipometer, respectively. The arm muscle circumference was calculated as arm muscle circumference (cm) = [arm circumference (cm) − (0.314 × tricipital skinfold (mm)] [31].

Adherence to exercise programs was evaluated considering exercise program attendance and was defined as reaching at least 80% of the recommended or prescribed exercise sessions [32].

### Sample size

A total sample size of 32 participants was estimated (16 in each study group), considering an 80% power, 5% significance level, a correlation of 0.7 among repeated measures, and a small effect size *f* of 0.2 for the peak VO_2_. To compensate the 20% estimated participant loss or refusal rate, each group must was composed of 19 participants, totalizing 38 participants.

### Randomization

Random allocation was determined by 10 blocks of 4 participants (Software Rx64 version 3.1.1), generated by an external researcher. Allocation concealment was implemented through a central randomization routine conducted by investigators with access to the randomized list and the investigator charged with requesting the code to place subjects in the intervention group.

### Statistical analyses

Descriptive statistical analysis using mean and standard deviation was used initially, followed by testing of normality by the Shapiro–Wilk test. Fisher’s exact test, Yates’ chi-squared test and Student’s t-test were used to compare the groups at baseline. Differences within groups (mean and standard deviations) and between groups (mean and 95% CI) were assessed before and after the 12-week intervention period. Generalized estimation equations were used for both analyses, followed by the Bonferroni’s post hoc test. The intention-to-treat analysis was performed with all randomized participants for the primary and secondary outcomes. The per-protocol analysis was performed for the same outcomes with participants who were classified as adherent to exercise programs (adherence ≥ 80%). Finally, the effect size of the differences within and between groups, in intention-to-treat analysis, was interpreted using Cohen's d statistic. An effect size < 0.2 reflects a negligible difference, ≥ 0.2 but < 0.5 a small difference, ≥ 0.5 but < 0.8 a moderate difference, and ≥ 0.8 a large difference [33]. All data were analyzed using SPSS Statistics for Windows version 20.0 (IBM Corp., Armonk, NY, USA). In all tests, a significance level of *p* < 0.05 was adopted.

## Results

**Fig. 1:**
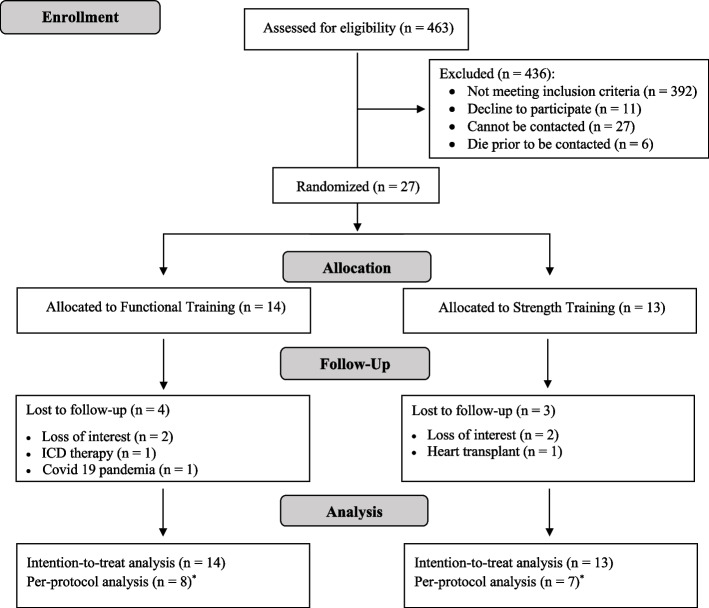
Design and flow of participants throughout the clinical trial. ICD: Implantable Cardioverter-Defibrillator. ^*^Only participants who were classified as adherent (adherence ≥ 80%)

In total, 27 participants were randomized into the experimental group, FTG (*n* = 14), or the active comparator group, STG (*n* = 13). The calculated sample size of 38 participants was not obtained because the trial was interrupted due to the COVID-19 pandemic. The recruiting and follow-up ranged from October 2017 to February 2020. Figure 1 shows the flow of participants in the study. Considering adherence to training programs, attendance rates were similar between groups: 72 ± 33% versus 72 ± 27% for the FTG and the STG, respectively.

After randomization and before the final assessment, four participants in the FTG and three participants in the STG withdrew from the intervention and final assessment of their clinical outcomes. In the FTG, the withdrawals occurred due to loss of interest (*n* = 2), implantable cardioverter-defibrillator procedure (*n* = 1), and restrictions imposed by COVID-19 (*n* = 1). In the STG, the withdrawals happened due to loss of interest (*n* = 2) and heart transplant (*n* = 1). Baseline values of clinical outcome measures for the patients who discontinued their study participation were included in the intention-to-treat analysis.

Baseline characteristics of participants are described in Table 1. Overall, participants were aged 60 years, mainly men, Caucasian, and overweight or obese. Non-ischemic cardiomyopathy was the most common etiology for HF while hypertension was the most prevalent comorbidity, followed by diabetes. All participants were taking beta-blockers—and most of them, angiotensin-converting enzyme inhibitors or angiotensin II receptor blockers. There were no baseline differences between groups except for active smoking (p = 0.043), that was more prevalent in the FTG than in the STG.

**Table 1:** Baseline demographic and clinical characteristics of the participants

Characteristic	Total sample (*n* = 27)	FTG (*n* = 14)	STG (*n* = 13)
Age (years)	60 ± 7	63 ± 4.9	58 ± 8.2
Men	18 (67)	9 (64)	9 (69)
Ethnicity			
Caucasian	19 (70)	11 (79)	8 (62)
Black	6 (22)	3 (21)	3 (23)
Other	2 (7.4)	0 (0)	2 (15)
BMI (kg/m^2^)	29 ± 6.3	29 ± 6.0	30 ± 6.8
Heart failure etiology			
Ischemic	13 (48)	8 (57)	5 (38)
Non-ischemic	14 (52)	6 (43)	8 (62)
NYHA			
II	21 (78)	11 (79)	10 (77)
III	6 (22)	3 (21)	3 (23)
LVEF	29 ± 8.5	30 ± 9.6	28 ± 7.4
Comorbidities			
Hypertension	15 (56)	6 (43)	9 (69)
Diabetes	14 (52)	6 (43)	8 (62)
ICD/CRT	8 (30)	4 (29)	4 (31)
Osteomuscular disorders	6 (22)	3 (21)	3 (23)
Active smoking	4 (15)	4 (29)	0 (0)*
COPD	3 (11)	1 (7.1)	2 (15)
Drug Therapy			
Beta-blockers	27 (100)	14 (100)	13 (100)
Diuretics	26 (96)	13 (93)	13 (100)
ACEI/ARB	23 (85)	12 (86)	11 (85)
Antiplatelet and/or anticoagulation agents	19 (70)	10 (71)	9 (69)
Statins	16 (59)	10 (71)	6 (46)
Digoxin	13 (48)	7 (50)	6 (46)
Nitrates	8 (30)	3 (21)	5 (38)

Data are expressed as means ± SD or n (%). Comparisons (*FTG*, Functional training group *vs, STG* Strength training group), *ACEI* Angiotensin-Converting Enzyme Inhibitor, *ARB* Angiotensin II Receptor Blocker, *BMI* Body Mass Index, *COPD* Chronic Obstructive Pulmonary Disease, *CRT* Cardiac Resynchronization Therapy, *ICD* Implantable Cardioverter-Defibrillator, *LVEF* Left Ventricular Ejection Fraction, *NYHA* New York Heart Association. *p < 0.05

The intention-to-treat analysis showed an improvement in peak VO_2_ (p group = 0.988, p time = 0.011, and p interaction = 0.921) and quality of life (p group = 0.067, p time = 0.001 and p interaction = 0.921) in both study groups after 12 weeks. Peak VO_2_ increased similarly by 1.4 ± 3.2 and 1.5 ± 2.5 mL.kg^−1^.min^−1^ in functional and strength training groups, respectively. The quality of life score decreased by 13.7 ± 15.0 points in the FTG and 12.0 ± 28.1 points in the STG. Considering the functionality, the intention-to-treat analysis showed an improvement in DASI in both study groups after 12 weeks of training (p group = 0.482, p time = 0.019, p interaction = 0.947); the FTG and the STG increased 6.5 ± 12.1 and 5.2 ± 13.2 points. The gait speed also increased in both groups after 12 weeks of training (p group = 0.913, p time = 0.002, p interaction = 0.576); the FTG and the STG increased 0.2 ± 0.3 m/s. The intention-to-treat results are shown in Table 2.

**Table 2:** Values of outcomes in each group, within groups, and between groups

	**Intention-to-treat analysis**						
**Outcomes**	Groups				Difference within groups		Difference between groups
	Week 0		Week 12		Week 12 minus Week 0		Week 12 minus Week 0
	FTG (*n* = 14)	STG (*n* = 13)	FTG (*n* = 14)	STG (*n* = 13)	FTG	STG	FTG minus STG
**Primary outcomes:**							
Peak VO_2_ (mL.kg^−1^.min^−1^)	16.9 (2.9)	16.8 (4.0)	18.6 (4.8)	18.6 (5.5)	1.4 (3.2)^*^	1.5 (2.5)^*^	0.12 (-2.5 to 2.7)
Quality of life score (points)	25.8 (14.8)	33.8 (23.8)	10.3 (7.8)	19.0 (15.1)	-13.7 (15.0)^*^	-12.0 (28.1)^*^	1.7 (-19.4 to 22.8)
**Secondary outcomes:**							
Duke activity status index (points)	28.8 (12.4)	26.2 (13.2)	34.5 (12.3)	33.0 (11.1)	6.5 (12.1)^*^	5.2 (13.2)^*^	-1.3 (-13.2 to 10.6)
Gait speed (m/s)	1.6 (0.3)	1.5 (0.2)	1.7 (0.3)	1.8 (0.3)	0.2 (0.3)^*^	0.2 (0.3)^*^	0.05 (-0.3 to 0.4)
Hand grip strength (kg)	31.5 (10.3)	28.7 (9.4)	28.4 (9.1)	32.4 (9.4)	-2.7 (5.5)	1.9 (4.8)	4.6 (-0.2 to 9.4)
Maximal inspiratory pressure (cmH_2_O)	64.5 (31.2)	66.1 (32.3)	63.5 (31.0)	56.2 (11.5)	1.6 (33.0)	-10.2 (21.8)	-11.8 (-38.1 to 14.5)
Endothelial function:							
Flow-mediated dilation (%)	6.3 (5.5)	9.8 (3.9)	4.8 (5.1)	6.4 (4.4)	1.2 (9.5)	3.4 (5.1)	2.2 (-5.8 to 10.3)
Nitroglycerine-induced vasodilation (%)	11.8 (5.0)	17.2 (5.8)	14.1 (7.6)	16.1 (9.0)	1.5 (8.1)	-0.8 (11.4)	-2.3 (-13.0 to 8.3)
Arm muscle circumference (cm)	29.6 (3.7)	28.8 (4.4)	29.6 (6.9)	29.1 (4.0)	1.3 (5.8)	0.9 (2.6)	-0.4 (-5.1 to 4.2)

Mean (SD) values for study outcomes in each group, mean (SD) difference within groups, and mean (95% CI) difference between groups

*FTG* Functional training group, *STG* Strength training group, *VO*_*2*_, Oxygen consumption

^*^Differences within groups after the 12-week intervention period (*p* < 0.05)

We observed similar results in the per-protocol analysis (see Supplemental Table 1). Peak VO_2_ (p group = 0.716, p time < 0.001 and p interaction = 0.895) and quality of life (p group = 0.354, p time = 0.013 and p interaction = 0.924) improved in both study groups after 12 weeks of training. Peak VO_2_ increased 2.6 ± 2.5 and 2.7 ± 2.0 mL.kg^−1^.min^−1^ in the FTG and the STG, respectively. The quality of life score decreased by 13.9 ± 9.8 points in the FTG and 12.8 ± 29.2 points in the STG. The per-protocol analysis showed an improvement only in gait speed in both study groups after 12 weeks of training (p group = 0.477, p time = 0.005, p interaction = 0.349); the FTG and the STG increased 0.2 ± 0.3 and 0.3 ± 0.4 m/s, respectively.

The effect size of the differences within and between groups in intention-to-treat analysis was calculated for peak VO_2_, quality of life, and functionality, as shown in Table 3.

**Table 3:** Effect size of the differences within and between groups in intention-to-treat analysis

	Intention-to-treat analysis		
Outcomes	Effect size within groups		Effect size between groups
	Functional Group (*n* = 14)	Strength Group (*n* = 13)	
Peak VO_2_ (mL.kg^−1^.min^−1^)	-0.486 (-1.033 to 0.078)	-0.654 (-1.245 to -0.041)	0.008 (-0.747 to 0.763)
Quality of life score (points)	0.950 (0.301 to 1.574)	0.541 (-0.053 to 1.115)	0.055 (-0.701 to 0.809)
Duke activity status index (points)	-0.538 (-1.091 to 0.033)	-0.587 (-1.168 to 0.014)	0.039 (-0.717 to 0.793)
Gait speed (m/s)	-0.662 (-1.233 to -0.070)	-0.681 (-1.276 to -0.063)	0.157 (-0.601 to 0.912)

Cohen's d statistic

Given the difference of 3.5 ml/kg/min (or 1 MET, metabolic equivalent), which we consider clinically relevant, the estimated power of the study is 71.3%. When we calculated the power using a difference of five points, which is considered clinically relevant for quality of life, the estimated power of the is 13,6%.

There were no differences within and between groups in the intention-to-treat and per-protocol analyses for peripheral muscular strength, maximal inspiratory pressure, endothelial function, and lean body mass.

All participants completed the study without any serious adverse events. In the FTG, there was only one episode of low back pain reported. Non-serious adverse events in the STG included hypoglycemia (*n* = 1), gout exacerbation (*n* = 3), and nausea (*n* = 3).

## Discussion

To our knowledge, this is the first randomized clinical trial aimed at evaluating the effects of functional training in individuals with HF. We compared functional training to strength training due to similarities between both physical modalities and because the latter is traditionally recommended by the American College of Cardiology Foundation and the American Heart Association as a non-pharmacological therapeutic approach in HF [34].

Even though adherence to both physical trainings was slightly below the recommended rate of 80%, our study demonstrated an improvement on peak VO_2_, quality of life, and functionality after 12-week functional and strength trainings. Many factors may affect exercise program adherence in individuals with HF, such as exercise setting, age, gender, race, NYHA class, left ventricle ejection fraction, and length of exercise duration. In our context, participants’ difficulties in commuting to the clinical research center because of distance and costs may have impacted on adherence to physical training programs.

Sperlich et al. [35] found an increase in peak VO_2_ of overweight, but apparently health women, after nine weeks of both high-intensity functional training alone (CircuitHIIT) or in combination with low-intensity functional training (Circuitcombined). CircuitHIIT increased peak VO_2_ by 10,1%, but with more perception of pain, while Circuitcombined increased only 4%. Considering the quality of life, Circuitcombined increased perception of general health to a greater extent than CircuitHIIT, probably because there was no enhanced perception of pain. Despite the differences related to population studied and some aspects of training prescription—HF patients and 12-week moderate-intensity functional training—our trial showed similar results. After the period of physical interventions, we also demonstrated an improvement of 10% in peak VO_2_. According to total score of quality of life, a clinically meaningful change ≥ 12 points was observed in both study groups. In addition, a moderate to large effect size within groups demonstrates the effectiveness of functional and strength training in improving the quality of life in these patients. Although we did not use a specific tool for pain assessment, patients were asked about the occurrence of adverse events and the presence of discomfort resulting from physical training. Currently, great importance has been given to the evaluation of patient-reported outcomes, and among the methods used for this evaluation are the quality-of-life questionnaires. For this reason, it is possible that there is a relationship between lower occurrence of symptoms related to the disease or to a given intervention and higher quality of life scores.

In a cross-sectional study, Schmidt et al. [10] demonstrated significant association between cardiorespiratory fitness, dynamic balance, and mobility with quality of life in individuals with HF with preserved ejection fraction. The authors showed that only dynamic balance and mobility were independently associated with total score, physical, and emotional dimensions of quality of life. Interestingly, the upper body strength (assessed by hand grip) and free fat mass (measured by bioelectrical impedance), were not associated with quality of life. Although our study did not evaluate the association between the variables and considering the methodological differences between the studies, our results corroborate these findings, as we demonstrated an improvement on peak VO_2_, functionality, and quality of life, but not on strength muscle and lean body mass. In fact, the DASI provides a standardized assessment of functional status that is significantly correlated with peak VO_2_, and uses the patient’s ability to perform a set of common activities of daily living to gauge functional capacity. As such, the responses can also be used to assess physical limitations relevant to the patient’s quality of life [25]. We believe that the increase in functionality has promoted quality of life.

Considering the functionality, the intention-to-treat analysis showed a meaningful improvement on DASI in both study groups after 12 weeks of training, but not in the per-protocol analysis. The moderate effect size within groups shows that functional and strength training are effective in improving functional status. We believe that the smaller sample size in the per-protocol analysis was the reason why we failed to detect differences in functional status between groups in this part of the analyses, since it includes only those individuals who have completed the treatment as planned. Gary et al. [7] assessed the effects of a 12-week progressive home-based program of moderate-intensity aerobic and resistance exercise on the physical function of adults with systolic HF. Although the authors found an increase in the 10-Item Continuous Scale Physical Functional Performance test, used to evaluate physical function, the DASI showed no change. The differences found between the study by Gary et al. [7] and our findings may be related to the initial physical activity status of the participants (49.2 versus < 35 points, respectively). We hypothesize that patients with worse physical activity status at baseline may respond to the intervention more powerfully. Despite the differences in the study population and the measurement of daily activities in the study by Vreede et al. [36]—healthy older women and Assessment of Daily Activity Performance scale, the authors also demonstrated that a 12-week training program of functional-task exercise and resistance exercise were both effective in improving functional task performance.

The present study also demonstrated interesting results in functionality related to gait speed. Aartolahti et al. [37] assessed the effects of long-term once-weekly strength and balance training on muscle strength and physical functioning in a community-based sample of aged adults. The authors found an improvement of 0.08 m/s in a 10-m walking speed test. Krebs et al. [38] showed a threefold gait speed improvements in a 6-week functional training compared with strength training performed by older adults. Our findings demonstrated a meaningful change of 0.2 m/s in a 5-m walking speed test in both study groups. The walking distance has varied between 3 and 10 m, although the distance has little effect on measured speed [39]. The 5-m distance has been adopted by large registries as it allows patients to achieve a steady walking speed without eliciting cardiopulmonary symptoms. The meaningful improvements in gait speed are estimated at 0.05 to 0.2 m/s [40]. The moderate effect size for the differences within groups demonstrated that both interventions are effective in increasing gait speed.

In our study, the functional training was performed with a strength component and did not change muscular strength. The systematic review performed by Liu et al. [8] examined the effects of functional training on muscular strength, physical functioning, and daily activities in older adults. Despite a large variability of exercise prescriptions, studies included in this systematic review demonstrated that functional training relates to improved mobility and daily activities. Muscle strength increased when functional training was performed with a strength component. When functional training was performed without a strength component, the findings did not favor functional training. These results may indicate a superiority of strength training over functional training in the field of muscle strength, but not over the ability to perform daily activities [8], reinforcing the principle of specificity of training, that is, training in a specific activity is the best way to maximize the performance in that specific activity [41]. Although strength exercises enhance muscular strength and exercise capacity, allowing for improvement in physical function and quality of life, no differences on muscle strength were found after 12 weeks of strength training. However, results showed that an improved functionality occurred in part due to changes in the peripheral muscle. The training of the muscles in the lower part of the body led to an improvement on the gait speed with repercussions on the functional status. Nevertheless, it was not possible to detect any changes in strength muscle due to the measuring tool that was used. In addition, the dose–response relationship between training intensity and gains in strength and physical functioning shows that high intensity training would be more effective than low intensity training [42]. Thus, it is possible that the intensity of strength training used in the present study was not sufficient to elicit changes in muscle strength and lean body mass.

Our study has limitations. First, the absence of a control group makes it difficult to identify changes in outcomes resulting from the intervention. However, including a usual care group without any exercise intervention is discouraged by the Research Ethics Committee, since it is notorious that physical exercise has positive effects in patients with HF. Second, our sample size was incomplete, because the study had to be finished earlier due to the COVID-19 pandemic. Finally, we believe that the measurement of lower-extremity muscle strength should also be done, since we prioritized lower-body exercises.

## Conclusion

Our study showed that functional training and strength are equally effective in improving oxygen consumption, quality of life, and functionality of individuals with HF. Our findings suggest that functional training may be a promising and innovative exercise-based strategy to treat HF. Future research is needed to better explore its effects in special subgroups of patients with HF.

## Supplementary Information

**Additional file 1:** **Supplemental Table 1.** Values of outcomes in each group, within groups, and between groups.

## Data Availability

We support the reuse of scholarly data and intend that the collected data in this trial may contribute beyond our actions to the knowledge on exercise and non-pharmacological management of HF. First, we provided in writing the final results of the research for each participant. Second, we have obtained ethical consent from participants as well as research ethics board approval to share deidentified data after trial completion through presentation in congresses and publications in journals. The datasets used and/or analyzed during the current study are available from the corresponding author on reasonable request.

## Abbreviations

DASI: Duke Activity Status Index
HF: Heart failure
VO_2_: Oxygen consumption
FMD: Flow-mediated dilatation
NYHA: New York Heart Association
