# Supplementary material for: Functional training improves peak oxygen consumption and quality of life of individuals with heart failure: a randomized clinical trial
Source: BMC Cardiovasc Disord. 2023 Jul 29;23:381. doi: 10.1186/s12872-023-03404-7 (PMC10386700; doi:10.1186/s12872-023-03404-7)
Supplement: Supplementary file 1 — Additional file 1: Supplemental Table 1. Values of outcomes in each group, within groups, and between groups. [file 12872_2023_3404_MOESM1_ESM.docx]

| **Supplemental Table 1** Values of outcomes in each group, within groups, and between groups. | | | | | | | | | | | | | | | | | | | | | | | | | |  |  |
| --- | --- | --- | --- | --- | --- | --- | --- | --- | --- | --- | --- | --- | --- | --- | --- | --- | --- | --- | --- | --- | --- | --- | --- | --- | --- | --- | --- |
|  |  | | **Per-protocol analysis** | | | | | | | | |  | |  |  | | |  | | |  | |  |  |  |  |  |
| **Outcomes** |  | | Groups | | | |  | |  | | | Difference within groups | | | | |  | | |  | | Difference between groups | | | | | |
|  |  | Week 0 | | | |  | | Week 12 | | | Week 12 minus Week 0 | | | | |  | | | Week 12 minus Week 0 | | | | | |  |  |  |
|  | FTG  (n = 8) | | STG  (n = 7) |  | FTG  (n = 8) | | | | | STG  (n = 7) | |  | FTG | | STG | | | | | | | FTG minus STG | |  | | |  |
| **Primary Outcomes:** |  | |  |  |  | | | | |  | |  |  | |  | | | | | | |  | |  | | |  |
| Peak VO_2_ (mL.kg^-1^.min^-1^) | 16.9 (3.1) | | 17.6 (4.8) |  | 19.4 (4.7) | | | | | 20.3 (5.5) | |  | 2.6 (2.5)* | | 2.7 (2.0)* | | | | | | | 1.2 (-2.4 to 2.7) | |  | | |  |
| Quality of life score (points) | 22.2 (12.0) | | 27.3 (28.0) |  | 8.4 (6.2) | | | | | 14.4 (10.2) | |  | -13.9 (9.8)* | | -12.8 (29.2)* | | | | | | | 1.0 (-22.6 to 24.6) | |  | | |  |
| **Secondary Outcomes:** |  | |  |  |  | | | | |  | |  |  | |  | | | | | | |  | |  | | |  |
| Duke activity status index (points) | 31.3 (11.1) | | 33.3 (13.5) |  | 34.3 (11.2) | | | | | 38.7 (5.5) | |  | 2.9 (9.8) | | 5.3 (15.7) | | | | | | | 2.4 (-11.9 to 16.7) | |  | | |  |
| Gait speed (m/s) | 1.5 (0.3) | | 1.6 (0.2) |  | 1.7 (0.3) | | | | | 1.9 (0,4) | |  | 0.2 (0.3)* | | 0.3 (0.4)* | | | | | | | 0.2 (-0.2 to 0.5) | |  | | |  |
| Hand grip strength (kg) | 29.4 (11.1) | | 29.6 (8.0) |  | 26.5 (8.3) | | | | | 31.6 (9.7) | |  | -2.9 (6.2) | | 3.0 (4.0) | | | | | | | -5.9 (0.02 to 11.7) | |  | | |  |
| Maximal inspiratory pressure (cmH_2_O) | 61.9 (38.6) | | 67.9 (25.2) |  | 67.1 (32.9) | | | | | 57.4 (10.4)) | |  | 5.2 (35.6) | | -10.4 (22.9) | | | | | | | 17.7 (-49.7 to 18.3) | |  | | |  |
| Endotelial function: |  | |  |  |  | | | | |  | |  |  | |  | | | | | | |  | |  | | |  |
| Flow-mediated dilation (%) | 6.6 (6.4) | | 9.3 (3.3) |  | 4.5 (5.8) | | | | | 5.4 (4.4) | |  | -2.1 (10.7) | | -3.4 (5.7) | | | | | | | 4.9 (-12.1 to 9.5) | |  | | |  |
| Nitroglycerine-induced vasodilation (%) | 12.0 (7.0) | | 15.7 (7.6) |  | 13.1 (8.7) | | | | | 13.5 (8.1) | |  | -0.2 (8.8) | | -3.5 (11.3) | | | | | | | -3.4 (-16.4 to 9.7) | |  | | |  |
| Arm muscle circumference (cm) | 27.7 (3.3) | | 28.2 (4.4) |  | 28.2 (5.8) | | | | | 28.7 (3.8) | |  | 0.6 (6.0) | | 0.5 (2.5) | | | | | | | -0.1 (-5.8 to 5.6) | |  | | |  |

Mean (SD) values for study outcomes in each group, mean (SD) difference within groups, and mean (95% CI) difference between groups. FTG: Functional training group; STG: Strength training group; VO_2_: Oxygen consumption. * Differences within groups after the 12-week intervention period (p < 0.05).
